# BioSphincters to treat Fecal Incontinence in Nonhuman Primates

**DOI:** 10.1038/s41598-019-54440-3

**Published:** 2019-12-02

**Authors:** Prabhash Dadhich, Jaime L. Bohl, Riccardo Tamburrini, Elie Zakhem, Christie Scott, Nancy Kock, Erin Mitchell, John Gilliam, Khalil N. Bitar

**Affiliations:** 1 Wake Forest Institute for Regenerative Medicine, Wake Forest School of Medicine, Winston Salem, NC USA; 2 Program in Neuro-Gastroenterology and Motility, Wake Forest School of Medicine, Winston Salem, NC USA; 3 Department of Surgery, Wake Forest School of Medicine, Winston Salem, NC USA; 4 Department of Pathology, Section on Comparative Medicine, Wake Forest School of Medicine, Winston Salem, NC USA; 5 Animal Resources Program, Wake Forest Baptist Health, Winston Salem, NC USA; 6 Section on Gastroenterology, Wake Forest School of Medicine, Winston Salem, NC USA; 7 Virginia Tech-Wake Forest School of Biomedical Engineering and Sciences, Wake Forest School of Medicine, Winston Salem, NC USA

**Keywords:** Tissue engineering, Faecal incontinence

## Abstract

Loss of anorectal resting pressure due to internal anal sphincter (IAS) dysfunctionality causes uncontrolled fecal soiling and leads to passive fecal incontinence (FI). The study is focused on immediate and long-term safety and potential efficacy of bioengineered IAS BioSphincters to treat passive FI in a clinically relevant large animal model of passive FI. Passive FI was successfully developed in Non-Human Primates (NHPs) model. The implantation of autologous intrinsically innervated functional constructs resolved the fecal soiling, restored the resting pressure and Recto Anal Inhibitory Reflex (RAIR) within 1-month. These results were sustained with time, and efficacy was preserved up to 12-months. The histological studies validated manometric results with the regeneration of a well-organized neuro-muscular population in IAS. The control groups (non-treated and sham) remained affected by poor anal hygiene, lower resting pressure, and reduced RAIR throughout the study. The pathological assessment of implants, blood, and the vital organs confirmed biocompatibility without any adverse effect after implantation. This regenerative approach of implanting intrinsically innervated IAS BioSphincters has the potential to offer a better quality of life to the patients suffering from FI.

## Introduction

Fecal Incontinence (FI) is the failure in maintaining anorectal pressure, resulting in involuntary leakage of stool and flatus. The loss of anal continence can be devastating from a social, psychological, and economic perspective. FI affects 2–6% U.S. population (aged 20–30 years), and this prevalence increases in older population (15%)^1^. Malfunctioning of the physiological parameters that control and maintain smooth muscle function, anorectal neural activity results in loss of internal anal sphincter (IAS) pressure and ultimately leads to passive FI^2^.

Standard of care management of passive FI is by adjustments of the diet, prescribing drugs that cause either diarrhea or constipation, attempts to bulk the stool, and biofeedback^3,4^. While these conservative measures may improve symptom severity in some patients. More aggressive approaches involve repeated delivery of inert biomaterials to improve the sphincter resistance^5^, but due to lack of bioactivity, the results were inconsistent and for a short duration of time. Anterior/posterior surgical repair has been tried, such as graciloplasty and sacral nerve stimulation^6,7^, but long-term results have been disappointing^7^.

There is a clinical demand for new therapies to address FI, particularly for patients who have severe low IAS pressure and passive incontinence. As per the NIH/NIDDK report, one third population reveal symptoms of fecal incontinence to health care providers. Regenerative medicine methods represent a personalized, customized, and novel approach to this debilitating disease. An autologous bioengineered Biosphincter proposed to repopulate cellular components and restore the structure of the IAS. This technology allows reinstatement of physiological IAS function and could treat FI.

Current approaches to treat FI are based on replenishment of striated muscle or mechanical support to external anal sphincter^8–11^. However, the terminal gut physiology is result of coordinated activity of rectum and IAS through synergistic functionality of smooth muscle cells with enteric neurons^12,13^. Therefore, restoration of neuronal and muscular components of IAS is essential for the reinstatement of continence. In previous studies, we developed a tissue-engineered substitute for cellular as well as functional restoration of IAS to remediate passive FI.

IAS constructs were engineered using smooth muscle cells (SMCs) and enteric neural progenitor cells (NPCs) isolated from gut biopsies^14,15^. The safety and integrity of engineered sphincters was evaluated via implantation into healthy animal model. The implanted sphincters were survived, vascularized, without any adverse effect, and maintained the physiological functionality^16–19^. The efficacy of engineered sphincters was evaluated via implantation of autologous engineered sphincters in rabbit passive FI model. The implantation of sphincters resulted in restoration of anorectal continence and reinstatement of basal pressure with Recto Anal Inhibitory Reflex (RAIR)^20,21^.

Rabbits are hindgut fermenters, thereby having more requirements for cecal digestion of food components, require a more fibrous diet, and are more susceptible to gastric ulceration and ileus than humans. The safety and efficacy of BioSphincters implantation need to be evaluated in a clinically relevant model. Non-Human Primates (NHPs) are mammalian and clinically relevant to humans. In this endeavor, a passive FI model of NHPs was developed, and intrinsically innervated autologous BioSphincters were implanted. Our hypothesis is to restore IAS function (reinstate basal pressure and RAIR) and treatment of fecal incontinence following implantation of autologous engineered BioSphincters. The primary objectives of the study were to (1) Develop a passive FI diseased model of NHP. (2) Evaluate long-term safety of autologous bioengineered innervated BioSphincters after implantation in the FI model. (3) Analyze the initial and long-term efficacy potentials of the treatment towards the reinstatement of IAS function and restoration of fecal continence.

It is the first report on the development of a clinical relevant non-human primate (NHP) model of passive FI. The study also describes the harvesting of IAS SMCs and NPCs from NHP. The isolation protocol for both cell types were standardized following good laboratory practice (GLP) standards. Innervated autologous BioSphincters were bioengineered and implanted (for 12-months) in passive FI model of NHP. A group of non-treated NHPs and sham NHPs were part of the study as a control. Safety and efficacy evaluated through physical, physiological, clinical, pathological, and histological parameters.

## Results

### Development of fecal incontinence

**Figure 1:**
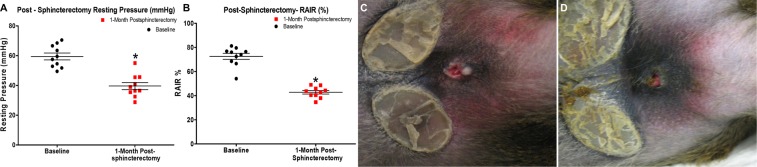
Development of passive FI following isolation of Cells from biopsies: (**A**) NHPs (n = 10) displayed a reduction in basal tone immediately after the hemi-sphincterectomy. (**B**) RAIR was reduced by 41% after sphincterectomy (n = 10); Fecal hygiene in NHP (**C**) before and (**D**) after hemi-sphincterectomy. *Values indicated p < 0.05 with Baseline; the horizontal bars exhibited mean and standard error.

All the NHPs were evaluated using rectal manometry to measure baseline resting pressure and RAIR of the IAS. The average resting pressure for all three groups (non-treated, treated, and sham) was 59.0 ± 2.2 mmHg (n = 10), and RAIR was 72.6 ± 2.5% (n = 10). The fecal output and hygiene were also normal. The hemi-sphincterectomy surgery severely affected the recto-anal physiology (p < 0.0001). Resting pressure was reduced to 39.5 ± 2.3 mmHg (n = 10), and RAIR decreased to 42.8 ± 1.4% (n = 10) in four weeks following hemi- sphincterectomy (Fig. 1A,B). These results were maintained in the non-treated group for 12 months.

Fecal material leaked from the anus and stained the perineum (Fig. 1C,D). There was fecal material scattered throughout the cage, which confirmed the uncontrolled release of stool. The reduction in anorectal pressure and fecal soiling corroborated the fecal incontinence. The body weight was unaffected, and none of the NHPs displayed any symptom of pain or agitation during this period.

### Bioengineering and characterization of autologous intrinsically innervated IAS sphincters

#### Isolation and characterization of smooth muscle cells

IAS biopsies (130 ± 15 µg, n = 6) were collected during the hemi-sphincterectomy. The viability of harvested cells was >96 ± 3%, as measured using NucleoCounter NC-200^TM^. Cells were characterized for the contractile phenotype of smooth muscle cells by positive staining with α-smooth muscle actin and smoothelin. The percentage purity of SMCs in the isolated cells was quantified using flow cytometry, where isolated cells were >95 ± 3 percent positive (n = 6) to smoothelin. The isolated cells displayed a positive and comparable expression of smoothelin compared to standard control confirming the SMCs population during qPCR.

All the isolations were successful, ~100 µg was the minimum biopsy weight required for successful isolation of IAS-SMCs. Isolated cells exhibited similar morphological and proliferation characteristics during all the isolations without any significant difference (p < 0.05; n = 6).

#### Isolation and characterization of neural progenitor cells

Jejunum biopsy (103 ± 18 µg, n = 6) was obtained from the small intestine via laparotomy. A modified technique of two-step digestion was adopted to isolate NPCs from harvested biopsies^20^. The isolated NPCs were cultured in non-treated flasks in a culture medium designed to enhance NPCs proliferation and avoid differentiation. The isolated NPCs were circular shaped cell bodies, proliferated in suspension condition, formed clusters resembling neurospheres like bodies. The viability of harvested cells was >93 ± 4%. The isolated NPCs were positive for the neural crest-derived cell marker p75^NTR^. During qPCR, the isolated cells displayed a positive and comparable expression of p75^NTR^ compared to standard control confirming isolation of NPCs population; these expressions were consistent up to five generations and reduced afterward. The cells were negative for the pluripotency markers Oct4 and smoothelin.

#### Bioengineering of autologous IAS sphincters

The isolated SMCs and NPCs from each NHPs were subcultured separately for five weeks to proliferate enough cells to engineer autologous IAS sphincters.

The optimized collagen hydrogel was mixed with 200,000 NPCs, and Sylgard coated plates. This hydrogel overlaid by another similar collagen hydrogel with 500,000 SMCs and incubated at 37 °C. During incubation, the collagen coagulated, and SMCs initiated orienting concentrically around the Sylgard post. Within 48 h, the cells were entirely contracted, and cell-hydrogel formed a ring-shaped sphincter. In this process, NPCs were also coming into close association with SMCs. The presence of SMCs enhanced the differentiation of NPCs into functional neurons. The axonal projection was protruding around the constructs by day six and formed a neural network throughout the bioengineered sphincter by day 12. Six BioSphincters were engineered, two were randomly used for cellular, structural, with functional analysis, and the remaining four were implanted.

#### Characterization of autologous IAS sphincters

**Figure 2:**
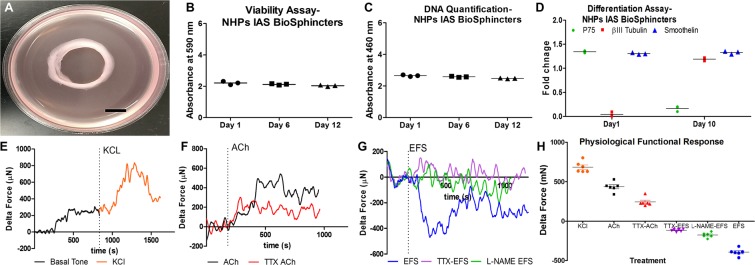
Bioengineering and characterization of IAS BioSphincters: (**A**) Bioengineered IAS BioSphincter. (**B**) Viability assay (n = 3); (**C**) Proliferation Assay (n = 3); (**D**) mRNA expression of different proteins at day 1 and 10; Physiological functional analysis exhibited; (**E**) generation of basal tone (black) following rapid contraction response to treatment with 60 mM potassium chloride (orange), which was maintained for 6 min and returned to baseline; (**F**) Rapid contractile response to 40 µM acetylcholine (black) treatment which partially diminished on pre-treatment of TTX (40 µM) in red; (**G**) Electrical field stimulation caused relaxation (blue) for 20 min, which was partially improved on pre-treatment of nNOS inhibitor (green) and completely attenuated on pre-treatment of TTX (40 µM); (**H**) summarized expression of physiological response exhibited by all the BioSphincters (n = 6) prior to implantation. This graph expressed the mean and standard error (n = 6) independent samples per group (p > 0.05); Line graphs show representative tracings to portray the trend of each treatment. Scale bar 5 mm in image A.

All the bioengineered sphincter constructs were completely contracted in a pre-defined 12-mm luminal diameter (Fig. 2A). The average surface area and volume of bioengineered sphincters were 10.25 ± 0.22 mm^2^ and 6.29 ± 0.15 mm^3^ (n = 6), respectively. The engineered IAS sphincter (n = 6) displayed 95.63 ± 3.0% and 92.63 ± 3.3% cell viability on day 6 and 12, respectively (Fig. 2B). The DNA quantity was also similar to day one without significant variation (Fig. 2C), further confirmed the permanence of viable and functional cells.

The longitudinal sections of fixed, paraffin-embedded bioengineered sphincters displayed several layers of cells in a reticulated network. These sections were positive for caldesmon and smoothelin. The mesh-like structures were also positive for primary antibodies directed against βlll tubulin and negative to primary antibodies directed against p75, which indicates differentiation of seeded NPCs towards functional neurons. The results were verified in negative controls. These results confirmed the development of physiologically relevant sphincters of IAS smooth muscle cells innervated with neuronal networks.

The qPCR analysis of neural differentiation normalized to housekeeping genes GAPDH, and 18 S ribosomal RNA revealed up-regulation of βlll tubulin from day 6 to day 10 and remained consistent up to day 14. Simultaneously, there was a decline in p75^NTR^ expression during the bioengineering of the constructs (Fig. 2D). The smoothelin expression was consistent throughout the bioengineering process, which confirmed the contractile phenotype of the muscle cells.

The viable and intrinsically innervated BioSphincters were further analyzed for physiological functionality using an isometric force transducer. The average characteristic spontaneous basal tone generated by BioSphincters was 250 ± 40 µN. The contractility and maturation of smooth muscle cells were tested. The addition of KCl (60 mM) caused a significant contraction above established basal tone, with a maximal average contraction of 684 ± 14 µN (n = 6) (Fig. 2E). KCl-induced contraction depends on membrane depolarization, which mediates through voltage-dependent Ca^2+^ channels present on muscles^22^. This significant contraction confirmed muscle contractibility in the sphincters. The effect of an excitatory neurotransmitter (Ach;  40 µM) resulted in an average contraction of 435 ± 25 µN (n = 6), which was significantly inhibited by 45% upon pre-treatment with a potent neurotoxin (TTX; 40 µM) (Fig. 2F). Ach-induced contraction mediates through muscarinic receptors present on SMC (M2R and M3R) and neurons (M1R)^14^. These results demonstrated that both the neuronal and muscular components were functional.

Neuronally evoked relaxation was tested by electrical field stimulation (EFS) (5 Hz, 0.5 ms), where BioSphincters exhibited relaxation to −395 ± 21 µN (n = 6). Pre-incubation with *N*_ω_-nitro-l-arginine methyl ester hydrochloride (L-NAME) (300 µM, neuronal nitric oxide synthase (nNOS) inhibitor), attenuated the relaxation response by ~70% which indicated the presence of inhibitory nitrergic neurons (Fig. 2G). The relaxation was completely abolished on pre-treatment with the neurotoxin (TTX; 40 µM) (Fig. 2G). These experiments confirmed the presence of a functional inhibitory motor neuronal population in the BioSphincters (Fig. 2H).

### Implantation and restoration of fecal continence

#### Fecal soiling and clinical investigation

The IAS hemi-sphincterectomy disturbed fecal hygiene as evidenced by fecal material dispersed throughout the cages. There was a lack of anal area hygiene as the perineum was always covered with a thin layer stool.

**Figure 3:**
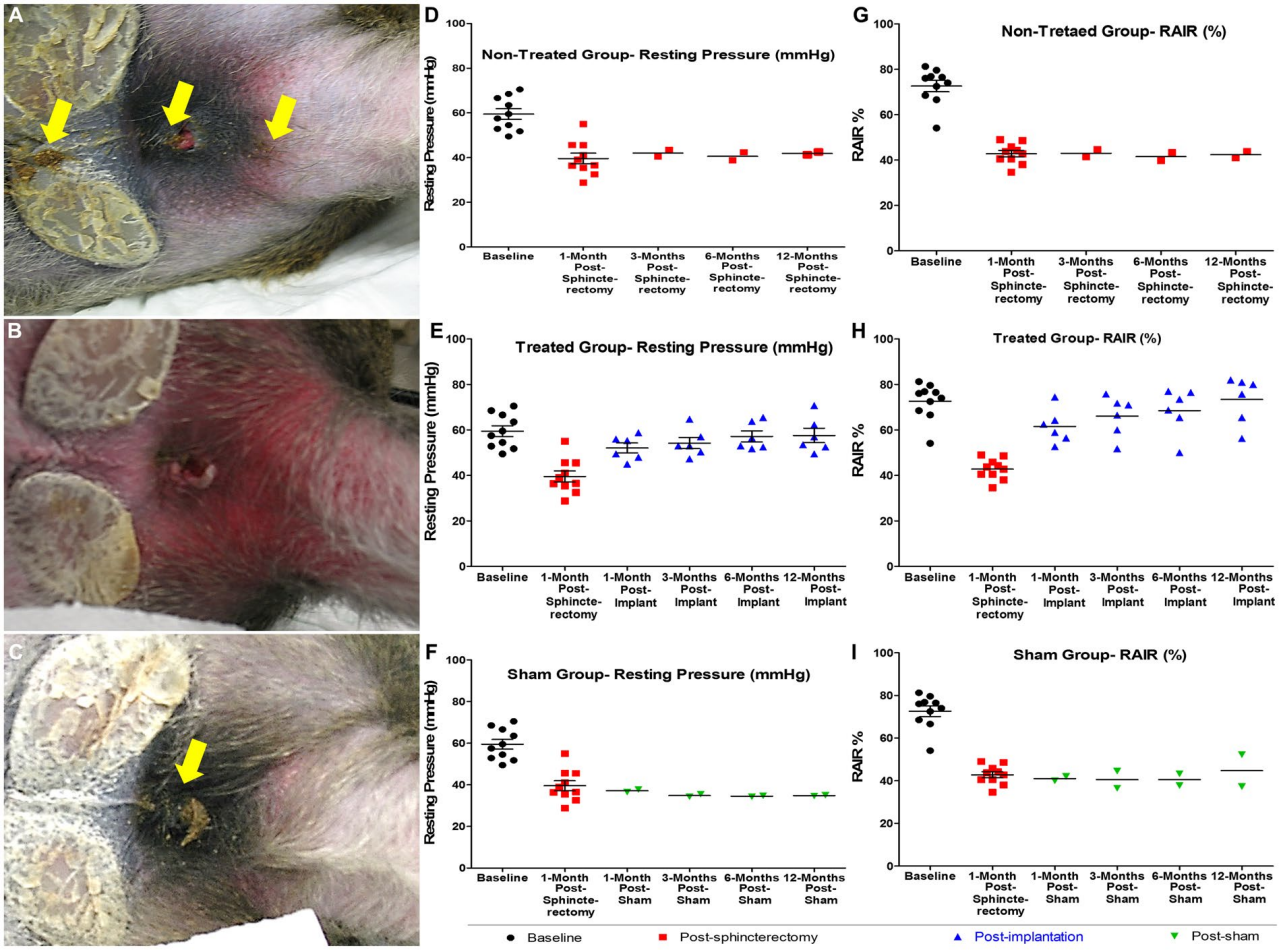
Post-implantation *in-vivo* efficacy of IAS BioSphincters: Fecal hygiene of (**A**) non-treated NHPs displayed fecal soiling (yellow arrows) and staining of perineum; (**B**) after implantation, fecal soiling resolved within one month and perineum was clean; (**C**) sham group was unaffected and displayed sustained fecal output (yellow arrow); (**D** and **G**) IAS hemi-sphincterectomy (n = 10) resulted in a significant reduction in anal basal pressure and RAIR compared to baseline, which was sustained to 1, 3, 6 and 12 months (n = 2); (**E** and **H**) The reduction in anal basal pressure and RAIR compared to baseline was restored within 1 month of implantation (n = 6), further improved and preserved for next 12 months (n = 6); (**F** and **I**) The anal basal pressure and RAIR were remained low and unaffected in the sham group (n = 2) throughout the study. The horizontal bars exhibited mean and standard error.

The fecal soiling and leakage of stool stopped 3–4 weeks after implantation and maintained throughout the study (12 months) (Fig. 3B). The fecal hygiene returned to normal in the treated group with the clean anal area and normal defecatory output compared to the non-treated group and sham group (Fig. 3A,C, respectively).

None of the NHP from any group exhibited any variation in physiological characteristics such as body temperature, body weight, muscle wasting, or/and atrophy. NHPs remained healthy without any adverse event. The weight of the NHPs was reported regularly, at baseline, pre/post surgeries, and before anorectal manometry. The NHPs in all three groups gained weight throughout the study without any adverse event (see supplemental material). The blood was collected from each NHP at a different time interval. The complete blood count and blood chemistry results exhibited no significant variation between different groups and were in a normal healthy range (see supplemental material).

#### *In vivo* efficacy

Anorectal manometry was carried out prior to any surgery. These measurements represented as baseline for all animals in this study. Anorectal manometry was further performed one month following IAS hemi-sphincterectomy (biopsy) and then at 1, 3, 6, and 12 months in each study group (Tables 1 and 2). In this way, each NHP acted as its own control.

**Table 1:** Summary of anorectal manometry readings (Basal pressure as mean ±SEM in mmHg) in all groups at different time points. As graphical representation Non-treated group displayed in Fig. 3D; Treated Group in 3E; and Sham Group in 3 F.

Baseline n = 10	1 month post-sphincterectomy n = 10	Non-treated group n = 2	1 month post-sphincterectomy	3 months post-sphincterectomy	6 months post-sphincterectomy	12 months post-sphincterectomy
			39.5 ± 2.3	42.0 ± 1.5	40.5 ± 1.8	41.9 ± 0.62
		Treated group n = 6	1 month post-implant	3 months post-implant	6 months post-implant	12 months post-implant
59.0 ± 2.2	39.5 ± 2.3		52.1 ± 2.1	54.2 ± 2.4	57.2 ± 2.6	59.2 ± 3.1
		Sham group n = 2	1 month post-sham	3 months post-sham	6 months post-sham	12 months post sham
			37.1 ± 5.6	34.9 ± 0.6	34.5 ± 0.2	34.8 ± 0.2

**Table 2:** Summary of anorectal manometry readings (RAIR as mean ± SEM in%) in all groups at different time points. As graphical representation Non-treated group displayed in Fig. 3G; Treated Group in 3H; and Sham Group in 3I.

Baseline n = 10	1 month post-sphincterectomy n = 10	Non-treated group n = 2	1 month post-sphincterectomy	3 months post-sphincterectomy	6 months post-sphincterectomy	12 months post- sphincterectomy
			42.8 ± 1.4	43.0 ± 1.5	41.5 ± 1.7	42.4 ± 1.3
		Treated group n = 6	1 month post-implant	3 months post-implant	6 months post-implant	12 months post-implant
72.6 ± 2.5	42.8 ± 1.4		61.5 ± 3.0	66.1 ± 3.6	68.6 ± 4.1	73.4 ± 4.2
		Sham group n = 2	1 month Post-sham	3 months Post-sham	6 months Post-sham	12 months post-sham
			41.0 ± 1	40.5 ± 4	40.5 ± 2.7	44.7 ± 7.5

#### Basal pressure

Hemi-circumferential internal anal sphincterectomy resulted in a significant decrease in basal tone. Basal pressure decreased from 59.0 ± 2.2 mmHg (n = 10) to 39.5 ± 2.3 mmHg at 1-month (n = 10), 42.0 ± 1.5 mmHg at 3 months (n = 2) post-sphincterectomy (Fig. 3D). Basal pressures at 1 and 3 months post-sphincterectomy were not considerably different from each other. In the non-treated group (n = 2), basal pressure remained low over the study period (40.5 ± 1.8 at 6 months and 41.9 ± 0.62 at 12-months).

After implantation, basal pressure significantly increased (52.1 ± 2.1 mmHg, n = 6) in the treated group (p < 0.005; Fig. 3E; Table 1) compared to basal pressure following sphincterectomy (39.5 ± 2.3 mmHg, n = 10). It was further sustained and improved at 3-months (54.2 ± 2.4 mmHg, n = 6), 6-months (57.2 ± 2.6 mmHg, n = 6), 12 months (59.2 ± 3.1 mmHg, n = 6) and restored to baseline (p > 0.05, n = 6).

In the sham group, basal pressure was significantly reduced from 59.0 ± 2.2 mmHg (baseline) to 37.15 ± 5.6 mmHg at the 1-month time point, 34.9 ± 0.6 mmHg at the 3-month time point (n = 2) (Fig. 3F). Basal pressure at 1 and 3 months post-sham was not noticeably different from basal pressure at 1 and 3 months post-sphincterectomy and remained low for the rest of the study period (Table 1). The basal pressure for the sham group was significantly lower than basal pressure post-implantation at 1, 3, 6, and 12 months (p < 0.05).

#### RAIR

Sphincterectomy also induced a significant decrease in RAIR in all NHPs. Post-sphincterectomy, RAIR significantly (p < 0.0001) decreased from 72.6 ± 2.5% (baseline, n = 10) to 42.8 ± 1.4% at 1 month (n = 10) (Fig. 3G), and remained low for following 3, 6 and 12 months’ time points (Table 2).

Following implantation of engineered BioSphincters, RAIR was restored to baseline (Fig. 3H) and was found to be significantly higher than RAIR post-sphincterectomy (p < 0.0001, n = 6). RAIR in the treated group increased to 61.5 ± 3.0 at 1 month, 66.1 ± 3.6% at 3 months (n = 6), 68.6 ± 4.1% at 6 months (n = 6) and 73.4 ± 4.2% at 12 months (n = 6).

In the sham group, RAIR was significantly reduced from 72.6 ± 2.5% (baseline) to 41.0 ± 1% at 1-month post-sham (n = 2), 40.5 ± 4.0% at the 3-month time point (n = 2) (Fig. 3I). This trend remained consistent in the following 6 and 12 months (Table 2). It was significantly lower than RAIR at 1, 3, 6, and 12 months post-implantation (p < 0.05). Multiple posthoc power analyses were performed based on both the basal pressure and RAIR parameters, using the Post-hoc Power Calculator tool available at (https://clincalc.com/stats/Power.aspx). Three months post sphincterectomy procedure, mean ± SEM values (basal pressure and RAIR), and sample sizes of the ‘non-treated’ and ‘treated groups’ were used to obtain the statistical power of the study. The alpha error (Type I error) value was set as 0.05 during the tests. During the tests, posthoc statistical power analyses were observed to be >90%, which further confirms the acceptability of the observations in the study with the given sample size.

#### Histological and pathological analysis

BioSphincters were harvested after 12 months of implantation. BioSphincters appeared intact around the anal canal. The BioSphincters displayed firm binding to the host tissue and was similar in dimension to the native tissue, which indicated little or no degradation of implanted BioSphincters. The presence of distinct blood vessels and the absence of avascular fibrous capsule surrounding to implant indicated no foreign body reaction.

**Figure 4:**
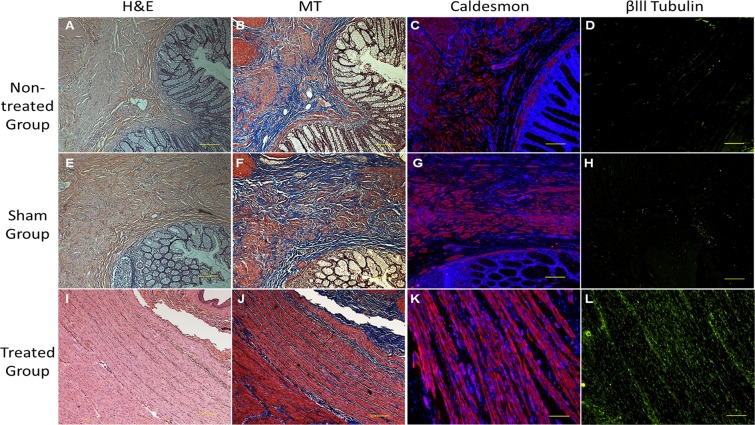
Post-implantation histological analysis of IAS BioSphincters: The H&E stained surgical site section of NHP in both (**A**) non-treated and (**B**) sham group displayed irregular and discontinued arrangement of cells and extracellular matrix, but (**C**) 12-months post-implantation sections displayed regular arrangment of cells; MT stained sections of (**D**) non-treated group and (**E**) sham group confirmed random distribution of muscle cells (red) in collagen (blue), (**F**) 12-months post-implantation sections exhibited highly aligned and uniformly distributed muscle cells (red) in collagen matrix (blue); The immunostaining with caldesmon further confirmed the lack of muscle cells in both (**G**) non-treated group and (**H**) sham group, whereas (**I**) treated group displayed well-arranged muscle network; A poor neuronal network was appeared on immunostaining with βlll Tubulin on (**J**) non-treated group and (**K**) sham group compared to dense and innervated neuronal network in (**L**) treated group. Scale bar 500 µm (in A,B,E,F,I,J) and 100 µm (in C,D,G,H,K,L).

The hematoxylin-eosin (H&E) (Fig. 4A,E) and Masson’s Trichrome (MT) (Fig. 4B,F) stained sections of non-treated and sham groups exhibited lack of muscles within the extracellular matrix. The immunohistochemical expressions for caldesmon and βlll tubulin were also weak for these groups (Fig. 4C,G,D,H). The loss of muscles validated low basal pressure and RAIR recordings during manometry in the non-treated and sham groups.

The H&E stained cross-sections of the implants in the treated group envisaged a thick, uniform continuous sheet of IAS tissue (Fig. 4I). It was further confirmed with MT staining, where muscles (stained in red) were distinctively aligned and interspersed in collagen (stained in blue) (Fig. 4J). The immunohistochemical analysis further confirmed thick bands of smooth muscle cells stained positive against the caldesmon antibody (Fig. 4K), innervated within the reticulated neuronal network, which stained positive against βlll tubulin (Fig. 4L). The formation of continuous, regular, and innervated neuro-muscular sphincter around the anal canal restored the basal pressure and RAIR as recorded in manometry.

Pathologic findings in the study were minor, consisted primarily of a non- significant incidence of background changes and not attributable to implantation. There was no evidence of neoplasia. Details of pathology methods and findings of the individual NHPs are attached as supplemental material.

## Discussion

Healthy anorectal physiology and functionality depend on the coordinated activity of the anal canal, EAS, and IAS. FI is also one of the complex dysfunctionality caused by diverse etiology and  lead to distressing psychosocial impact on the quality of life^13^. In passive FI, due to an abnormality of IAS, the anal sphincter has lost normal response to signals that the rectum is full. It leads to leakage of stool, mucus, and flatus without awareness. It is reported that the IAS maintains the basal tone of the anal canal and responsible for ~70% of resting pressure and RAIR. The complex interplay between SMCs and neural network are responsible for relaxation and contraction, which allows feces to pass and anal tone to be re-established^23,24^. However, the symptoms and severity of passive FI can be heterogeneous across the patient population despite similar cellular abnormalities. Typically, IAS dysfunction results from a marked reduction in both SMCs and neural cell population or loss of integrity of the IAS muscle cells and its intrinsic enteric innervation without any identified cause.

The injection of bulking agents, biofeedback, and sacral nerve stimulation provide brief symptomatic relief, but the roots of the disease remain unaddressed^7^. In addition, the efficacy of these methods varies according to an individual’s training, experience, and type of treatment protocol that remains non-standardized. Therefore, a regenerative medicine approach is an advanced treatment avenue to target the FI at the cellular level with the host’s own tissue. In this context, previous attempts that were made using stem cells resulted in varied outcomes^8,25,26^. Direct stem cell injection in FI rats resulted in higher anorectal pressure compared to native pressure. The optimal route and carrier of cell delivery is another challenge with this approach. The carrier will act as stem cell-niche, which will affect cell differentiation and may cause adverse effects. In another approach, myogenic stem cells were injected alone, and with biogel in EAS resulted in improvement EAS functionality^11,27^. In the advancement of bulking agents delivery, autologous myoblasts were injected with polycaprolactone beads and resulted in improved anal sphincter function, but this study was lacking control groups to investigate the effect of cells alone^28^. Safety, viability, and long-term cell differentiation is still unaddressed with these methods. These treatment options are either palliative solutions and inadequate for the long-term relief with uncertain safety and efficacy of results for passive FI. The autologous intrinsically innervated BioSphincters was proposed as an alternative therapy to substitute the deficient cellular population and reinstate the functionality for passive FI.

To evalute the long-term safety and efficacy of the proposed BioSphincter approach, there is a need of an adequate animal models of dysfunctional IAS, which leads to passive FI. In previous reports, the sphincterectomy caused insignificant loss of IAS, resulted in short-term (~14 days) loss of resting and peak anal pressure after sphincter injury^29,30^. In another report, EAS and IAS were both resected via quadrant sphincterectomy and displayed a sustained reduction in anorectal pressure but lead to FI through the heterogeneous loss of both IAS and EAS function, where passive FI is mainly caused by loss of integrity of IAS^31^.

Our group targeted resection of only IAS without affecting EAS, which resulted in an immediate drop of anorectal pressure and RAIR, which was sustained for at least three months^17,20^. A similar approach was used in the present study to create a passive FI NHP model. The manometric recordings post-sphincterectomy confirmed a significant reduction in pressure (n = 10). Quantitatively, there was reduction in basal pressure (39.5 ± 2.3 mmHg; 33%; n = 10) and a drop in RAIR (42.8 ± 1.4%; 41%; n = 10) post-sphincterectomy. In both the non-treated and sham groups, this reduction in physiological function did not recover and remained low throughout the 12 months’ observation period. Histological analysis corroborated these findings. It also confirmed lack of self-regeneration of IAS tissues. There was no evidence of possible fibrosis or scarring at the surgical site in the sham or non-treated group. These results confirmed the successful development of the passive FI model in all the ten NHPs. The hemi-sphincterectomy procedure resulted in a reproducible and sustained animal model of passive FI, which is clinically analogous to the human.

The harvested IAS biopsy was processed to isolate smooth muscle cells using previously standardized protocol. Neural progenitor cells were isolated from small intestine biopsies. Both types of isolated cells were mixed individually with laminin and type-1 collagen to form the hydrogel. The collagen formed the hydrogel and acted as support 3D matrix for the cells. Laminin is essential for functional neural cells differentiation and innervation of bioengineering BioSphincters. SMCs have a unique ability to restructure collagen hydrogel and align circularly owing to matrix metalloproteinase activity. At the same time, NPCs differentiated into functional neurons and innervated mature SMCs during bioengineering process in the development of sphincters. The engineered Biosphincter of circularly oriented muscle cells with innervations mimic and substitute the degenerated IAS tissue. We have optimized the process to conform to the diameter of bioengineered tissue to different sizes as needed.

The bioengineered IAS BioSphincters exhibited a stable basal tone and did not display any spontaneous phasic contractile movement, which confirmed their tissue specificity and functionality as IAS. The pre-and post-implant BioSphincters exhibited voltage-dependent Ca2+ channels mediated contraction upon stimulation with KCl and evidenced effective electromechanical coupling in the muscles. ACh response further confirmed cholinergic-induced contraction, myogenic, and neuronal characteristics of BioSphincters. This response was reduced on pre-treatment with TTX, indicating the exogenous addition of ACh might have induced the release of endogenous neurotransmitters from the intrinsic innervation of BioSphincters, increased the magnitude of contraction due to neuro-muscular response. These responses were consistent post-implantation, as well.

The basal pressure is responsible for maintaining the closure of the anal canal, and relaxation of BioSphincter is equally essential for defecation. The pre- and post-implanted BioSphincters exhibited relaxation of enteric innervation upon direct EFS stimulation.

These results were corroborated with previous *in vitro* studies of human IAS strips^32^. This mechanism was further confirmed when relaxation was abolished by pre-treatment with TTX. In another set of experiments, the magnitude of EFS-induced response was significantly (~70%) reduced during pre-incubation with NO- blocker LNAME. The results on pre-and post-implanted BioSphincters were similar and confirmed the stable and robust ability of relaxation of BioSphincters through nNOS neurons in BioSphincters. After *in vitro* testing for viability, orientation, differentiation, and physiological functionality, the engineered autologous BioSphincters using autologous cells were implanted approximately 6–8 weeks post-biopsy procedure.

The first objective of the implantation was to investigate the safety of the implanted engineered sphincters. The blood parameters, body weight, and physical parameters were extensively recorded and analyzed during the study. The animals remained healthy and gained weight as usual in all the groups. The detail clinical analysis of blood was normal in all the three groups, without any significant variation from baseline. The pathological evaluation of the implantation site and other vital organs after 12 months of implantation exhibited no adverse effects such as tumorigenicity or neoplasia.

The second objective of the study was to evaluate the efficacy. The diminished basal pressure (39.5 ± 2.3 mmHg; 33%; n = 10) and diminished RAIR (42.8 ± 1.4%; 41%; n = 10) after sphincterectomy was restored up to 52.1 ± 2.1 (88%; n = 6) and 61.5 ± 3.0 (85%; n = 6), respectively within four weeks after implantation of autologous engineered BioSphincters in treated group. The basal pressure and RAIR completely reinstated to baseline within six months and remained stable up to the completion of the study. There was no significant difference in the basal pressure and RAIR recorded as baseline and treated group (after implantation). The non-treated and sham groups did not display any improvement in the manometric recordings and remained close to sphincterectomy level throughout the 12-months. Statistical comparison was not possible with non-treated and sham groups due to small group (n = 2).

Rectal distension during RAIR measurement causes a transient increase in anal pressure followed by symmetrical reduction of anal pressure caused by relaxation of IAS. This relaxation is also an indicator of the existence of functional nNOS neurons and the restoration of physiological integrity of the IAS. The reinstatement of RAIR to baseline indicated restoration of neuronal network, specifically repopulation and integration of nNOS-mediated inhibitory functional neurons of implanted BioSphincters with native host tissues. It was further corroborated with histological findings: cross-section of IAS of treated groups exhibited continuous reticulated neuronal network with aligned muscle population. The histological sections using immunostaining with muscle and neuronal antibodies displayed robust and organized cell orientation (Fig. 4).

We developed the innovative approach of bioengineering of intrinsically innervated NHP IAS BioSphincters using isolated NPCs and IAS-SMCs, which mimics the physiological functionality of native IAS tissue. The cells in these BioSphincters demonstrated characteristic phenotypes, muscular alignment, and differentiation. The bioengineered BioSphincters confirmed the physiological functionality via quantitative response to different pharmacological stimulants, and these responses were preserved after implantation over an extended period of time. This is the first pre-clinical study of tissue-engineered products towards the treatment of passive FI in NHPs, which is most relevant to human subjects. The primate pre-clinical study showed the long-term safety and efficacy of the BioSphincters. The BioSphincters approach provides an effective autologous treatment for patients with passive fecal incontinence.

## Conclusion

### Development of FI model

Passive FI was successfully induced in all the NHPs by hemi-sphincterectomy (180° resection of the anterior IAS sphincter), without any surgical complication. The animals defecated without any discomfort. Compared to pre-sphincterectomy, feces were leaked and scattered around the perineum. External signs of FI were accompanied by changes in anorectal manometry, including a reduction in (i) anal basal pressure, due to smooth muscle weakness and (ii) RAIR, owing to loss of neural control in the IAS. These parameters are consistent with passive FI in humans.

### Bioengineering and implantation of BioSphincters

SMCs and NPCs were isolated and characterized from IAS and jejunum biopsies, respectively. Intrinsically innervated IAS BioSphincters were bioengineered from the isolated autologous cells and were successfully implanted in the respective animal. The bioengineering process was optimized to tailor customized BioSphincters of different diameters. There was no indication of any rectal obstruction, anal stenosis, or adverse event reported due to implantation.

### Safety and efficacy evaluation after implantation

The treated group resumed normal defecatory bowel movement without fecal soiling within three weeks of implantation. The non-treated group and sham group exhibited no improvement in fecal hygiene.

The BioSphincters implanted animals exhibited reinstatement of stable basal tone and RAIR during anorectal manometry. Compared to sham and non-treated groups, manometry readings in the treated group confirmed that the bioengineered BioSphincters were functional after implantation and restored both the muscle and neural components. Animals were monitored up to 12 months after implantation. Histological studies confirmed the stability and preservation of implanted cell types as intact as one circular tissue integrated with native tissues. Histopathology analysis validated biocompatibility and safety of the implantation without any fibrosis and neoplasia. The animals had healthy weights and blood count.

The large animal pre-clinical study in the NHP model exhibited long-term safety and efficacy of the autologous interracially innervated BioSphincters to treat passive FI. This new innovative approach will reinstate continence and offer a better quality of life to the patients by providing an additive functional intrinsically innervated IAS bioengineered from their own cells.

## Methods

### Study design

Ten NHPs (cynomolgus, male, 7–10Kg, 6years) were enrolled in the study. Anorectal manometry (resting pressure and Recto Anal Inhibitory Reflex, RAIR) was performed at the initiation of the study, followed by two surgeries. In the first surgery, fecal incontinence was induced by hemi-sphincterectomy of IAS and excised IAS was used to isolate SMCs. Jejunum biopsies were also obtained in the same surgery to isolate NPCs. NHPs were categorized into three groups (Table 3): (i) non-treated group, (ii) treated group, (iii) sham group.

**Table 3:** Experiment design.

	Enrolled NHPs	Baseline manometry	First Surgery (to induce FI)	Manometry post-sphincterectomy	Second Surgery	Each month up to 12 months
Non-treated group IAS hemi-sphincterectomy only	2	√	√	√	—	Manometry
Treated group IAS hemi-sphincterectomy followed by implantation of BioSphincters	6	√	√	√	Implantation	
Sham group IAS hemi-sphincterectomy followed by sham surgery	2	√	√	√	Sham surgery	

The BioSphincters were bioengineered from isolated cells. Quality control (sterility, viability, and functionality) and structural properties were validated.

6 to 8 weeks post biopsy; a second implantation surgery was performed on the NHPs of the treated group. There was a sham second surgery for the non-treated group. Manometry, anal hygiene, body weight, and blood analysis were performed up to 12 months.

### Reagents

Reagents were obtained from Life Technologies unless specified otherwise. SMCs growth media consisted of DMEM high-glucose, 10% FBS, and 2 mM l-glutamine. NPCs growth media consisted of neurobasal, 1xN2 supplement, recombinant-human epidermal growth factor, recombinant-basic fibroblast growth factor. BioSphincter culture media consisted of neurobasal medium-A, 2% FBS, 1xB27 supplement. All the media supplemented with 1x antibiotic-antimycotic. Tetrodotoxin (TTX) and *N*_ω_-nitro-l-arginine-methyl-ester-hydrochloride (L-NAME) were purchased from Sigma. The anti-smoothelin antibody [R4A] is from Abcam (dilution 1:100; catalog no. ab8969). The anti-caldesmon antibody is from Abcam (5 µg/ml; catalog no. ab183146). The anti- alpha-smooth muscle Actin antibody [4A4] is from Abcam (dilution 1:200; catalog no. ab19952). The anti-p75 NGF Receptor antibody is from Abcam (1 µg/ml; catalog no. ab212684). The anti-beta III Tubulin antibody is from Abcam (10 µg/ml; catalog no. ab119100).

### Animal care

The animal experiments were reviewed and approved by the Institutional Animal Care and Use Committee (IACUC) at Wake Forest School of Medicine. The surgical procedure, post-operation care, analgesics, and monthly manometric measurements were performed in accordance with GLP guidelines.

### Development for FI

Passive FI was induced by hemi-sphincterectomy, as described previously^20^. Briefly, a curvilinear incision was made ventrally between the anal canal and IAS at the anocutaneous tissue. The IAS was amputated hemi-circumferential beneath the EAS in the submucosal plane (see supplementary material).

Antimesenteric biopsies were also collected from jejunum through laparotomy.

### Cell isolation and characterization

The cell isolation was optimized following previously described protocol^14^. The process of cell isolation, subculture, and engineering of BioSphincters were documented and reviewed as per the GLP guidelines.

#### IAS-SMCs

IAS biopsies were minced into ~1 mm pieces and washed three times with Hank’s Balanced Salt Solution (HBSS) wash solution (containing 2X antibiotic-antimycotics) prior to and after mincing. The finely minced biopsies digested twice for one hour, each with 1.0 mg/ml collagenase type-II (Worthington Biochemicals) solution in DMEM. The isolated cells were washed and cultured in SMCs growth media, followed by characterized via immuno-reactivity against smoothelin and α-smooth muscle actin markers.

#### Enteric NPCs

Jejunum biopsies were washed, minced, and followed by two-step digestion (containing 0.85 mg/ml type-II collagenase, 0.85 mg/ml dispase-II, and 20 µl/ml DNAse-1 in DMEM) at 37 °C an hour each. The digested tissue was washed and strained through 70-µm and 40-µm nylon cell strainer before plating. The isolated cells characterized by neural crest-derived cell marker p75.

In the isolation process, cell viability, proliferation analysis, contractile physiology (for SMCs) and stemness virtues (for NPCs), were quantified for different passages to standardize the isolation protocol for NHP species.

#### Bioengineering of BioSphincters

A previously described method^20^ was modified to bioengineer BioSphincters. Briefly, 200,000 NPCs/BioSphincter were mixed with 1xDMEM, 10% FBS, 0.8 mg/ml type-I collagen (BD Biosciences), 20 µg/ml laminin. The hydrogel was seeded uniformly around the post in sylgard coated plate. Similarly, another hydrogel mixture (except laminin) was prepared using 500,000 SMCs/BioSphincters and over-layered on the previous gel.

### Characterization of BioSphincters

#### Biological analysis

The BioSphincter was analyzed against multiple antibodies to characterize SMCs (caldesmon and smoothelin) and differentiation of NPCs (βlll tubulin and p75). The results were corroborated with protein expression via Quantitative polymerase chain reaction (qPCR). The GAPDH and 18s-ribosomal were housekeeping genes, whereas smoothelin, p75, beta-III tubulin, and nNOS were target genes. Resulting expressions were normalized with housekeeping gene, and fold change was calculated with the standard gene.

#### Functional analysis

Engineered BioSphincters were analyzed for physiological functionality prior and post-implantation. The BioSphincter hooked in a horizontal tissue bath (Harvard Apparatus) and data acquired using LabChart-7 software (AD Instruments).

The spontaneous basal-tone was measured. Effect of the excitatory (Acetylcholine (ACh; 40 μmol/l) and relaxant (electrical field stimulation; EFS; 5 Hz, 0.5 ms) stimulants were evaluated. Pre-treatment with TTX (1 μmol/l) and L-NAME (300 µM) was carried out to characterize neo-innervation.

The sphincters were implanted in the treated group. The pre-operative preparation and operative procedure were similar to the hemi-sphincterectomy procedure as described previosuly^20^.

### Safety and efficacy analysis

#### Anorectal manometry

As described previously^20^, for anal basal pressure, a catheter with circumferentially arranged four air-charged pressure transducers was inserted inside the anal canal and slowly withdrawn to identify and record the area of maximum resting pressure (basal pressure). During RAIR, the balloon attached at the end of the catheter was inflated to a volume of 30 ml, provided a percentage decrease in basal pressure in response to the internal pressure generated by balloon. Data acquisition and analysis were performed using BioVIEW software (see supplementary material).

#### Clinical evaluation and Histopathological analysis

Complete blood count (CBC), clinical blood analysis, weight, diet, physical health conditions of each NHP was assessed and documented at regular time points.

Histopathological analysis of BioSphincters and vital organs (see Supplemental Material) was carried out, where a board-certified veterinary pathologist examined hematoxylin-eosin (H&E) and Masson’s Trichrome (MT) stained sections in a blinded fashion. All lesions were identified by major process, location, distribution, and graded as minimal, mild, moderate, or marked.

### Statistical analysis

All the quantitative and semi-quantitative studies were statistically analyzed by two-tailed Student’s t-test, expressed as mean ± SE. One-way ANOVA was performed with Bonferroni post-hoc analysis to compare anal basal pressure and RAIR among groups. All authors had access to the study data, reviewed, and approved the final manuscript.

## Supplementary information

SUPPLEMENTARY INFO
