# Supplementary material for: BioSphincters to treat Fecal Incontinence in Nonhuman Primates
Source: Sci Rep. 2019 Dec 2;9:18096. doi: 10.1038/s41598-019-54440-3 (PMC6888838; doi:10.1038/s41598-019-54440-3)
Supplement: Supplementary file 1 — SUPPLEMENTARY INFO [file 41598_2019_54440_MOESM1_ESM.docx]

**BioSphincters to treat Fecal Incontinence in Nonhuman Primates**

Prabhash Dadhich^1,2^, Jaime L. Bohl^3^, Riccardo Tamburrini^3^, Elie Zakhem^1,2^, Christie Scott^1^, Nancy Kock^4^, Erin Mitchell^5^, John Gilliam^6^, Khalil N Bitar^1,2,6,7 *^

^1^Wake Forest Institute for Regenerative Medicine, Wake Forest School of Medicine, Winston Salem, NC, USA

^2^Program in Neuro-Gastroenterology and Motility, Wake Forest School of Medicine, Winston Salem, NC, USA

^3^Department of Surgery, Wake Forest School of Medicine, Winston Salem, NC, USA

^4^Department of Pathology, Section on Comparative Medicine, Wake Forest School of Medicine, Winston Salem, NC, US

^5^Animal Resources Program, Wake Forest Baptist Health, Winston Salem, NC, USA

^6^Section on Gastroenterology, Wake Forest School of Medicine, Winston Salem, NC, USA

^7^Virginia Tech-Wake Forest School of Biomedical Engineering and Sciences, Wake Forest School of Medicine, Winston Salem, NC, USA

**Address Correspondence to:**

*Khalil N. Bitar, PhD., AGAF

Wake Forest Institute for Regenerative Medicine Phone: (336) 713-7266

391 Technology Way FAX: (336) 713-7290

Winston-Salem NC 27101 E-mail: [kbitar@wakehealth.edu](mailto:kbitar@wakehealth.edu)

**Supplemental Material**

# **Histopathological analysis of BioSphincters and vital organs**

**Pathology Methods**

Species and Strain: Ten adult male cynomolgus macaques.

Tissues were collected at necropsy following a standardized protocol, and included:

| **Table 1: The type of tissues collected after necropsy for histopathological analysis** | |
| --- | --- |
| **Category** | **Tissue types** |
| **Surgical / Implantation Site** | Internal Anal Sphincter (IAS) and rectum, including the implant or sham surgical site or equivalent site |
|  | Adjacent Skeletal muscles |
|  | Skin |
|  | Anal Glands |
| **Gastrointestinal Tract** | Stomach |
|  | Duodenum |
|  | Small Intestine |
|  | Ileum |
|  | Cecum |
|  | Colon/Large Intestine |
| **Urinary Tract** | Kidney, left and right |
|  | Ureters |
|  | Bladder |
| **Vital Organs** | Heart (left and right ventricles) |
|  | Brain (1-4 sections) |
|  | Liver, 1-4 samples |
|  | Lung (left & right lobes) |
|  | Adrenal Glands |
|  | Any abnormalities if noted at the gross examination |

Tissues were promptly fixed in 10% neutral buffered formalin, embedded in paraffin, sectioned at 5 – 6µm, and stained with hematoxylin and eosin. In addition, 1-2 segments from the procedure site (IAS) from each animal were stained with Masson’s trichrome stain to assess fibrosis.

All sectioned tissues were examined by an American College of Veterinary Pathologists board-certified veterinary pathologist (NDK), and recorded, in a blinded fashion with respect to treatment group. All lesions were identified by the major process, location, distribution, and if possible time course, and graded as minimal, mild, moderate, or marked. All observations were recorded in an Excel spreadsheet. After unblinding, the findings were summarized below and tabulated in the table following.

**Histologic Findings**

**Surgical Site** (internal anal sphincter and rectum): Fibrosis variably replaced both the inner and outer muscular tunics of the rectum in all cases. The glandular loss occurred in 7 cases, and mucosal-associated lymphoid tissue was prominent in 4. The mucosa was thinned in 5 cases and ulcerated in 1. The inner muscular tunic wall was segmentally thickened in 6 cases, and the wall was segmentally thinned in 4. The remodeling of the rectal wall was considered an appropriate response to the surgical procedures with attempted healing.

**Gastrointestinal Tract:** (stomach, duodenum, ileum, colon, rectum): The lesions were mostly mild to moderate inflammatory lesions with gastritis in 6 cases and enteritis in 2. The mucosal-associated lymphoid tissue in the rectum of 6 animals was prominent (5 in the Spx+Imp group) but this was considered a physiologic process, representing a response to antigenic stimulation.

**Urinary Tract** (kidneys, ureter, urinary bladder, urethra): The ureters and urethras were considered within normal limits, except one in which the mucosa was ulcerated. Four animals had mild cystitis. The kidneys of one of the NHPs had moderate to marked granulomatous nephritis, part of a systemic process in this animal.

**Heart:**  Significant lesions were not identified in any of the sections examined. One animal had mild adipocyte infiltration and another minimal eosinophilic myocarditis.

**Liver:** Most of the lesions identified in the liver were mild and considered background or incidental. These consisted of the mild portal or multifocal inflammation, vacuolar degeneration (possibly stress-related), extramedullary hematopoiesis, and glycogenosis (most likely physiologic).

**Lung:**  One animal (8434) had pulmonary granulomas, which were part of a systemic process in this animal. Significant lesions were not identified, and any of the other sections examined, and all were mild. These included terminal airways smooth muscle hyperplasia, inflammation, fibrosis, arterial smooth muscle hyperplasia, prominent mucosal-associated lymphoid tissue, and pneumocyte hyperplasia.

**Pancreas:** Four animals had varying degrees of adipocyte infiltration, all of which were considered incidental lesions. A granuloma was present in one of the NHPs, the animal with the systemic granulomatous process.

**Spleen:** Significant lesions were not identified in any of the sections examined.

Two animals had reactive lymphoid tissue, a response to antigenic stimulation. One had lymphoid depletion, not an uncommon finding in laboratory primates, but possibly associated with immune compromise, as this was one of the NHPs, the animal with the systemic granulomatous disease.

A**drenal glands:** Significant lesions were not identified in any of the sections examined.

**Lymph nodes:** Significant lesions were not identified in any of the sections examined.

All lymph nodes had varying degrees of sinus histiocytosis reflective of tissue drainage from sites of inflammation, eight were considered reactive (response to antigenic stimulation), and one had sinus erythrocytosis reflective of tissue drainage from a site of hemorrhage.

**Prostate gland:** Significant lesions were not identified in any of the sections examined. Three animals had minimal inflammation (prostatitis), and one had glandular ectasia (possibly due to blockage distally). Prostate gland was not consistently present in all cases.

**Brain:** Significant lesions were not identified in any of the sections examined. One animal had an embolus from the bone marrow.

**Lesions noted at the gross examination:** What was identified as a lesion in the spleen in 8473 was a calcified nodule surrounded by a thick fibrous capsule, likely representing prior parasite infection.

**Conclusions:** The histologic findings in this study, aside from those at the procedure sites, were generally minor background changes, mostly inflammatory and not uncommon findings in laboratory primates. Some changes were considered physiologic (lymphoid hyperplasia, hepatocellular glycogenosis, lymph node sinus histiocytosis), and therefore, not true lesions. Foreign material was not identified in any of the sections examined, and special stains for acid-fast bacteria (*Mycobacterium* spp.) and fungi were negative. Because the inflammation had an eosinophil component, the parasitic infection was also considered, but agents were not identified in the sections examined.

1. **Weights of the NHPs:**

NHPs were weighed regularly, at baseline, on the days of surgeries and post surgeries. Weights are presented in bar graphs (Figure 1). NHPs maintained healthy weights and gained weights throughout the study period.


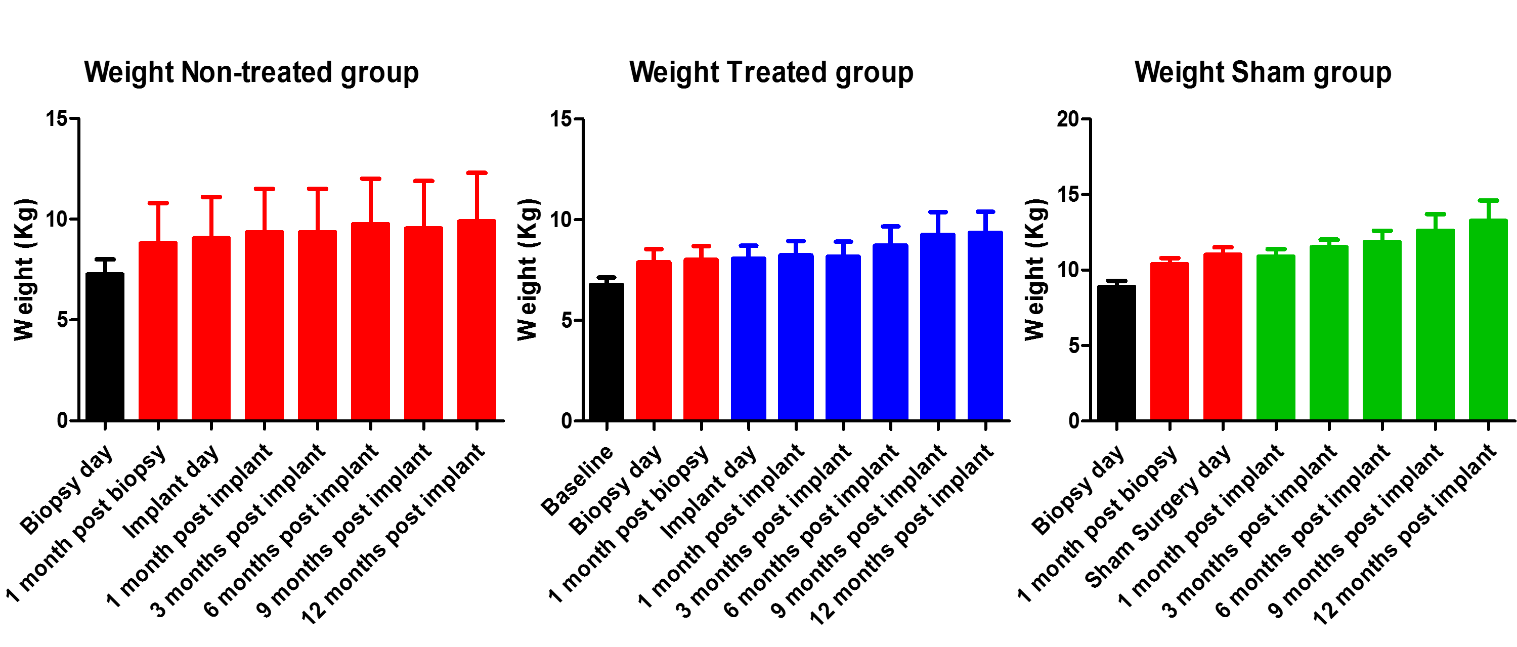


**Figure 1. Weight trend.** NHPs gained weight consistently throughout the study. There is an increasing trend in the weight in all groups. Weights remained within the normal range.

1. **Blood Results:**

Venous blood samples were taken from the femoral vein of NHPs at baseline and then at the defined time-points. The samples placed on wet ice and delivered to a contract laboratory (IDEXX) same day. Results are shown in the following table 2. Blood was analyzed and compared among different groups. Blood results were given to Dr. Erin Mitchell, DVM who analyzed and interpreted the results as discussed in table 2.

**Table 2: Means and standard error of the mean of the blood results**

| **Parameters** | **Baseline** | | **Treated Group (n=4)** | | **Non treated Group (n=2)** | | **Sham Group (n=2)** | |
| --- | --- | --- | --- | --- | --- | --- | --- | --- |
|  | ***Mean*** | ***Std. Error*** | ***Mean*** | ***Std. Error*** | ***Mean*** | ***Std. Error*** | ***Mean*** | ***Std. Error*** |
| **ALP (UL)** | 89.60 | 10.16 | 80.67 | 11.97 | 64.50 | 5.50 | 82.50 | 15.00 |
| **AST (UL)** | 54.30 | 9.64 | 48.00 | 6.92 | 46.00 | 12.00 | 67.50 | 9.25 |
| **ALT (UL)** | 35.10 | 6.82 | 72.67 | 15.50 | 36.00 | 7.00 | 47.00 | 9.62 |
| **Creatine Kinase (UL)** | 744.70 | 173.30 | 578.33 | 146.98 | 627.50 | 318.50 | 3312.00 | 133.91 |
| **GGT (UL)** | 45.30 | 3.52 | 48.50 | 6.75 | 52.50 | 5.50 | 47.00 | 9.46 |
| **Amylase (UL)** | 192.60 | 22.83 | 210.50 | 34.52 | 149.00 | 1.00 | 180.50 | 32.80 |
| **Lipase (UL)** | 17.60 | 3.09 | 32.33 | 13.38 | 12.50 | 2.50 | 21.50 | 4.19 |
| **Albumin (gdL)** | 3.84 | 0.16 | 3.92 | 0.09 | 3.55 | 0.05 | 3.40 | 0.70 |
| **Total Bilirubin (mgdL)** | 0.31 | 0.05 | 0.20 | 0.00 | 0.20 | 0.00 | 0.25 | 0.05 |
| **Total Protein (gdL)** | 6.82 | 0.15 | 6.95 | 0.21 | 6.50 | 0.00 | 6.35 | 1.31 |
| **Globulin (gdL)** | 2.98 | 0.15 | 3.03 | 0.29 | 2.95 | 0.05 | 2.95 | 0.58 |
| **BUN (mgdL)** | 14.90 | 0.84 | 12.33 | 1.58 | 10.00 | 3.00 | 11.00 | 1.82 |
| **Creatinine (mgdL)** | 0.93 | 0.10 | 1.02 | 0.07 | 0.80 | 0.00 | 0.85 | 0.17 |
| **Cholesterol (mgdL)** | 104.90 | 7.79 | 111.83 | 6.60 | 107.00 | 21.00 | 102.50 | 20.68 |
| **Glucose (mgdL)** | 47.50 | 5.22 | 118.50 | 36.82 | 111.50 | 58.50 | 242.50 | 30.40 |
| **Calcium (mgdL)** | 9.13 | 0.15 | 9.57 | 0.22 | 8.90 | 0.40 | 8.70 | 1.75 |
| **Phosphorus (mgdL)** | 5.01 | 0.18 | 4.23 | 0.72 | 4.30 | 0.20 | 3.85 | 0.78 |
| **Chloride (mmoL)** | 107.11 | 0.72 | 107.50 | 1.06 | 106.50 | 2.50 | 106.50 | 21.23 |
| **Potassium (mmoL)** | 5.04 | 0.28 | 4.90 | 0.21 | 5.10 | 0.70 | 6.20 | 1.05 |
| **WBC** | 11.51 | 1.74 | 11.43 | 1.06 | 13.60 | 3.10 | 14.65 | 2.69 |
| **RBC** | 5.57 | 0.13 | 5.85 | 0.20 | 5.76 | 0.16 | 5.47 | 1.14 |
| **HGB** | 13.14 | 0.28 | 14.00 | 0.45 | 13.40 | 0.30 | 13.25 | 2.70 |
| **HCT** | 42.32 | 0.63 | 44.07 | 1.51 | 43.70 | 0.80 | 43.50 | 8.69 |
| **Platelet Count** | 297.90 | 36.23 | 361.83 | 37.89 | 344.50 | 44.50 | 360.00 | 65.78 |
| **Neutrophil** | 6041.50 | 1154.89 | 6003.67 | 642.11 | 6906.00 | 2496.00 | 7364.50 | 1323.23 |
| **Lymphocyte** | 4460.70 | 556.54 | 4310.17 | 477.06 | 4882.00 | 262.00 | 5498.00 | 1016.28 |
| **Monocyte** | 795.00 | 143.45 | 836.00 | 193.37 | 1548.00 | 256.00 | 1464.00 | 286.40 |
| **Eosinophil** | 196.90 | 50.30 | 269.33 | 146.79 | 251.00 | 83.00 | 309.00 | 40.44 |
| **Basophil** | 16.70 | 3.94 | 14.00 | 6.93 | 14.00 | 3.00 | 14.50 | 2.29 |

1. **Post-Implantation Physiological Analysis:**

After euthanasia after 12-month time point, the IAS was harvested from all the NHPs (non-treated, sham, and treated) and tested in organ bath for the physiological response. After the stable basal tone, the smooth muscle cell contractility (as average maximum force) on the addition of KCl (60mM) was measured as 654 ± 17 µN (n=6), 330 ± 10 µN (n=2), 310 ± 15 µN (n=2) in treated, non-treated and sham group, respectively. This response confirmed significantly higher (n<0.05) integrity and functionality of muscles in the treated group, compared to passive FI model. The neuronal evoked contraction was studied using acetylcholine (Ach; 1µM; an agonist-induced excitatory stimulation). The treated group exhibited 428 ± 8.8 µN force, which was significantly higher (n<0.05) than non-treated group (222 ± 12 µN) and sham group (202 ± 7.5 µN), respectively. Similarly, the neuronal evoked relaxation response was measured following EFS treatment. These results confirmed the reinstatement of contraction compared to the diseased model. The IAS of the implanted group displayed robust relaxation with average maximum force during relaxation was -403 ± 6 µN for the implanted group, -245 ± 15 µN in the non-treated group and -252 ± 9 µN in the sham group. The contractile response to agonist-induced contraction and EFS-induced relaxation after implantation was successfully evoked and confirmed that the differentiated neurons were physiologically active and reinstated the functionality. The formation of synaptic connections between injected neural cells and native tissue was detected through diminished response to ACh upon inhibition of neurons in the treated group. Which was absent in the non-treated group and sham group.

|  |  | **KCl** | **ACh** | **TTX-ACh** | **EFS** | **LNAME-EFS** | **TTX-EFS** |
| --- | --- | --- | --- | --- | --- | --- | --- |
|  |  |  |  |  |  |  |  |
| **Treated Group (n=6)** | **Force ± SE** | **654 ± 17** | **428 ± 8.8** | **223 ± 8.7** | **-403 ± 6** | **-187.5 ± 6** | **-110.8 ± 6** |
| **Non-Treated Group (n=2)** | **Force ± SE** | **330 ± 10** | **222 ± 12** | **200 ± 5** | **-245 ± 15** | **-200 ± 5** | **-167.5 ± 7** |
| **Sham Group (n=2)** | **Force ± SE** | **310 ± 15** | **202 ± 7.5** | **195 ± 10** | **-252 ± 9** | **-190 ± 5** | **-160± 5** |
|  |  |  |  |  |  |  |  |

1. **Hemi-circumferential sphincterectomy procedure:**

For the hemi-circumferential sphincterectomy, an enema was performed before the surgical procedure to remove feces in order to reduce contamination of the anal surgical site. A small diameter catheter was inserted in the NHP's anal canal. Up to 20 mL of water/saline was used for irrigation of the colon to remove any fecal pellets. The surgical site was aseptically prepared as best as possible and then draped into a sterile field. Two stay sutures were placed at approximately 3 and 9 o’clock positions near the anal verge for exposure. Along the anal verge at the 12 o’clock position, a single curvilinear incision was made above the dentate line through skin and dermis. Sharp and blunt dissection was providing exposure of the anal sphincter. The IAS was amputated hemi-circumferential beneath the EAS in the submucosal plane. The IAS was dissected, and ~1 cm of IAS tissue was excised. The skin was closed with interrupted 3-0 to 6-0 monofilament sutures in an inverted or simple interrupted pattern or subcuticular pattern. Simple interrupted sutures may also be placed as needed. Sutures were removed 7-14 days’ post-surgery under anesthesia.

1. **Implantation of BioSphincters:**

All procedures were performed aseptically. The animal was chemically sedated and then maintained. Ophthalmic ointment was applied. Up to two peripheral IV catheters were placed. Nails were clipped as necessary. An enema was performed before the surgical procedure to remove feces in order to reduce contamination of the anal surgical site. A small diameter catheter was inserted in the NHP's anal canal. Up to 20 mL of water/saline will be used for irrigation of the colon to remove any fecal pellets. The surgical site was aseptically prepared as best as possible and then draped into a sterile field. A space between the native IAS and external anal sphincters was created by dissecting a plane between the two anal sphincters. The bioengineered sphincters were placed into this space and secured using tissue glue and sutures as necessary. In the case of a sham procedure, no sphincter was placed. Incisions were closed with 3-0 to 6-0 monofilament sutures. Sutures were removed by 7-14 days post-operatively under anesthesia as needed.

1. **Manometry procedure:**

While the animal was sedated, anorectal manometry was performed to measure the pressure of Internal Anal Sphincter. Following sedation, the animal was laid on their right side, and the catheter was inserted into the rectum 7 cm deep. The catheter has four air-charged pressure transducers arranged at the same level circumferentially and 90° apart. The catheter was then withdrawn in 1 cm increments and the area of maximum resting pressure (anal basal pressure) was identified. Anal basal pressure was recorded. A balloon attached to the distal aspect of the catheter was used to evaluate RAIR, which is expressed as the percentage decrease in basal pressure in response to rectal balloon inflation to a volume of 20 mL. Data acquisition and analysis were performed using BioVIEW software (Sandhill Scientific, Littleton, CO). It was done pre-operatively. The procedure was repeated up to once per month until the animal's respective time point.
